# Insights from fifteen years of real-world development, testing and implementation of youth digital mental health interventions^☆^

**DOI:** 10.1016/j.invent.2025.100849

**Published:** 2025-06-27

**Authors:** Shane Cross, Shaminka Mangelsdorf, Lee Valentine, Shaunagh O'Sullivan, Carla McEnery, Isabelle Scott, Tamsyn Gilbertson, Shona Louis, Jon Myer, Ping Liu, Niel Mac Dhonnagáin, Tom Wren, Eleanor Carey, Daniela Cagliarini, Ross Jacobs, Roos Pot-Kolder, Imogen Bell, Jennifer Nicholas, Lucia Valmaggia, John Gleeson, Mario Alvarez-Jimenez

**Affiliations:** a Orygen, Parkville, Melbourne, Australia; b Centre for Youth Mental Health, The University of Melbourne, Melbourne, Australia; c Healthy Brain and Mind Research Centre, School of Behavioural and Health Sciences, The Australian Catholic University, School of Psychology, Melbourne, Australia

**Keywords:** Digital mental health, Youth, Implementation, Engagement, Co-design, Personalisation, Transdiagnostic, Blended care, Scalability, Large language models

## Abstract

This paper reviews the current evidence and synthesizes fifteen years of real-world development, testing, and implementation of digital mental health interventions (DMHIs) for young people. Drawing on the work of Orygen Digital, we outline the evolution of interventions including the Moderated Online Social Therapy (MOST) platform, the Mello app, and a suite of virtual reality-based therapies; all developed to meet the complex clinical, developmental, and service needs of youth aged 12 to 25.

We identify ten key challenges and opportunities encountered in designing, developing and implementing these DMHIs: (1) meaningful co-design; (2) sustained user engagement; (3) personalization and transdiagnostic targeting; (4) optimizing intensities of human support; (5) leveraging peer support and social networking; (6) embedding DMHIs in clinical services; (7) blending digital and face-to-face care; (8) building data infrastructure and learning health systems; (9) developing sustainable and scalable business models; and (10) preparing DMHIs for large language models. Each theme reflects both achievements and persistent challenges, and is illustrated through a synthesis of the current evidence and real-world insights from our clinical trials and national-scale service implementations.

Our approach is grounded in various frameworks including clinical staging, self-determination theory, supportive accountability, and minimally disruptive medicine. Emerging innovations such as just-in-time adaptive interventions, extended reality (XR) therapies, stratified treatment models, and large language models offer promising future pathways for greater personalization, engagement, effectiveness, and scalability.

Our findings highlight the potential value of context-sensitive, co-designed, and system-integrated DMHIs, while also emphasising enduring limitations such as variable engagement, implementation barriers, and population-specific adaptation. Moving beyond controlled efficacy trials toward agile, real-world learning health systems will be essential to realising the full potential of DMHIs in transforming youth mental health care.

## Highlights

• Reviews 15 years of real-world youth digital mental health intervention work
• Identifies 10 key challenge themes shaping design, delivery, and implementation of DMHIs
• Combines clinical trials with national-scale implementation experience
• Highlights engagement, service delivery and implementation frameworks
• Explores future directions including extended reality, large language models and learning health systems

## 1 Introduction

Mental ill health is the leading cause of disability among young people aged 12–25 years, (Gore et al., 2011) and a significant contributor to disease burden in many parts of the world (Patton et al., 2016). Evidence suggests that prevalence rates have risen over the last decade (McGorry et al., 2024). Mental illnesses have a peak onset around the age of 15 years, with an estimated 63–75 % of all disorder onsets occurring before the age of 25 years (McGorry et al., 2024). This is important because when disorders emerge during this stage of life, significant disruptions are observed in development and maturation, identity and relationship formation, educational and vocational attainment, financial independence and personal autonomy (McGorry et al., 2024). These disruptions can have lasting consequences for young people's physical, mental, emotional, and social health, limiting their ability to thrive and undermining their capacity to develop the support, confidence, and resources needed to navigate life's challenges and reach their full potential (World Health Organization, 2024; Ross et al., 2020)

Despite the substantial and increasing prevalence of mental ill health in this age range, youth mental health services remain under-resourced and therefore unable to meet demand (McGorry et al., 2024). Moreover, a substantial proportion of young people with mental ill health do not seek treatment (Whiteford et al., 2014). Mirroring findings from other high-income countries (Ghafari et al., 2022), recent Australian data indicates that 21.5 % of the population and 38.8 % of young people aged 16–24 years experienced a mental disorder in the previous 12-months. Among those affected, 45.1 % consulted a health professional, 21.3 % saw a psychologist and 4.8 % accessed a digital mental health service (Australian Bureau of Statistics, 2020). Systemic problems in youth mental health service provision, including long waits for services (Subotic-Kerry et al., 2024), low levels of uptake and engagement once in care (Seidler et al., 2020) and modest treatment effects (Weisz et al., 2023). Additional barriers include financial constraints, stigma deterring help-seeking, and significant workforce shortages, leading to provider burnout and reduced service quality (Aguirre Velasco et al., 2020). Notably, the majority of young people live in low- and middle-income countries where they face additional and unique socioeconomic and cultural challenges that impact on their mental well-being and ability to access timely care (Rose-Clarke et al., 2024).

### 1.1 Role of technology in improving mental health care for young people

Integrating digital technology into youth mental health services can help address these limitations and is now a national and international policy priority (Mohr et al., 2018; Productivity Commission, 2020), supported by evidence of cost-effectiveness in adult populations (Andersson et al., 2019). Most research and development over the nearly 30-year history of internet-delivered treatment has involved adult populations, and has demonstrated that digital mental health interventions (DMHIs) significantly enhance accessibility while achieving engagement and effectiveness outcomes comparable to face-to-face therapy (Andersson et al., 2019; Carlbring et al., 2018; Cross et al., 2022). Additionally, digital interventions offer the potential for scalable delivery, and substantially lower cost per person than traditional face-to-face services (Productivity Commission, 2020).

In contrast to adults, young people have distinct developmental needs, greater recency of illness onset and a significant proportion have high-risk severe illness trajectories (McGorry et al., 2022; van Os, 2013). Further, many young people are new to mental health care (Australian Bureau of Statistics, 2020; Lawrence et al., 2015), with limited knowledge of available services or their healthcare rights. As digital natives, they are generally motivated to use technology to support their mental health (Ball et al., 2019; Bell et al., 2022), yet require age-appropriate, personalised tools. Two recent reviews of 21 (Bergin et al., 2020) and 71 (Liverpool et al., 2020) youth DMHIs concluded that while these interventions show promise, more research is required to establish their real-world effectiveness and cost-effectiveness.

Over the past 15 years, our team has developed, evaluated, and implemented DMHIs for young people and youth mental health services. In the following section, we detail the DMHIs developed by our team and summarize the knowledge generated with each. We then go on to identify ten key challenges we have gleaned from the current literature and directly encountered in the development and implementation of our DMHIs identified through team-based reflection on recurring challenges and opportunities encountered in practice. External literature was prioritised if it was recent, peer-reviewed and either directly addressed youth populations or offered transferrable insights. Internal data and experiences were drawn from our published and in-press research, supplemented by real-world practice-based insights from delivering a real-world national digital mental health service. By synthesising the broader knowledge base with our own experiences, the aim of this article is to provide a comprehensive overview of the issues affecting the field, highlight critical gaps and share practice-based insights to inform future research, development and implementation efforts.

## 2 Orygen and Orygen Digital

Orygen, Australia's National Centre of Excellence in Youth Mental Health, has led research, service delivery, and policy for over 30 years. Its technology arm, Orygen Digital, develops and delivers a range of digital youth mental health products and services. The following sections describe the primary products and services our team has developed over the past 15 years.

### 2.1 Integrated digital mental health services - MOST

The Moderated Online Social Therapy (MOST) model was developed 15 years ago as a multi-component DMHI designed for specific populations and employed within research trials. Now in its fifth iteration, MOST is a flexible technology platform (defined as an integrated digital environment that hosts a range of digital features and services) that works alongside face-to-face services to offer bespoke therapeutic content, interaction with clinicians, career consultants and peer workers, and a moderated online social network (Cross et al., 2023b). Its adaptability allows for clinical service delivery across a range of disorders and treatment contexts.

The first pilot of MOST, ‘Horyzons’, involved 20 young people with first-episode psychosis (Alvarez-Jimenez et al., 2013) and later progressed to a randomised controlled trial (RCT) evaluating its efficacy during recovery from first-episode psychosis (Alvarez-Jimenez et al., 2021). Since then, versions of MOST with different therapeutic content for use in different clinical contexts have been evaluated in seven additional completed or ongoing RCTs (Alvarez-Jimenez et al., 2025, Alvarez-Jimenez et al., 2021; Mangelsdorf et al., 2024; O'Sullivan et al., 2022; Simmons et al., 2021) and 11 pilot studies across a range of clinical presentations, including borderline personality disorder (O'Sullivan et al., 2024), ultra-high risk for psychosis (Alvarez-Jimenez et al., 2018), depression (Rice et al., 2018), anxiety and depression (Alvarez-Jimenez et al., 2020; Rice et al., 2020), social anxiety (McEnery et al., 2019), borderline personality disorder (O'Sullivan et al., 2024), vocational recovery (Simmons et al., 2021), suicide risk (Bailey et al., 2020) and bipolar disorder (in trial). These interventions have also involved families and carers (Gleeson et al., 2021, Gleeson et al., 2023a, Gleeson et al., 2023b), span the treatment spectrum from early help-seeking to relapse prevention, and have been adapted internationally, including in the USA (Ludwig et al., 2020; Pokowitz et al., 2023), Canada (Lal et al., 2020, Lal et al., 2023), the Netherlands (van Doorn et al., 2023) and Ireland (Dwan-O'Reilly et al., in press).

Despite variation in therapeutic content across iterations, MOST has maintained a consistent conceptual framework and demonstrated high engagement, safety, and acceptability among young people (Cross et al., 2023b). Young people have reported that MOST fosters a sense of belonging and normalisation, reduces stigma, and supports help-seeking by offering a structured yet flexible digital environment for real-time peer connection and professional support (O'Sullivan et al., 2024; Valentine et al., 2021, Valentine et al., 2020). Studies have shown clinical effectiveness in reducing depression and anxiety, improving vocational outcomes, and halving hospitalisation and emergency service visit rates in youth psychosis, alongside strong cost-effectiveness and service endorsement (Alvarez-Jimenez et al., 2021; Engel et al., 2024). A detailed summary of outcomes across clinical trials and pilot studies including findings on effectiveness, acceptability, and service impact is provided in Supplementary Table 1.

These early trials laid the groundwork for scaling MOST within youth mental health systems, including a blended care pilot (eOrygen) integrating with face-to-face services (O'Sullivan et al., 2024). This pilot found MOST to be safe (no adverse events), feasible (low refusal rate of 25 %) and acceptable (93 % of young people would recommend to others) as a blended model, with clinicians endorsing the evidence-based therapeutic content on MOST. The COVID-19 pandemic acted as a critical inflection point, prompting significant investment from the Victorian State Government following its Royal Commission into Mental Health (Royal Commission into Victoria's Mental Health System, 2021). Drawing on prior implementation evidence, this funding enabled the rapid deployment of an updated MOST platform across the state's youth mental health services. With additional support from other state and federal agencies, MOST is now available in over 400 youth mental health services Australia wide.

The current version of MOST is a transdiagnostic, multi-component digital service (Cross et al., 2023b). It is designed to function as a first-line treatment option, whether accessed independently or via referral. When used within services it aims to address critical service gaps such as long wait times, lack of between-session support, and limited post-discharge care (Cross et al., 2023b; Lederman et al., 2014). A recent service evaluation showed strong engagement, high satisfaction, and promising clinical outcomes (Alvarez-Jimenez et al., 2024; Cross et al., 2025), and further evaluation via a new RCT is currently underway (Alvarez-Jimenez et al., 2025). As with many DMHIs, observed challenges have included sustaining user engagement over long time periods, integrating MOST into the clinical workflows of external services and determining the optimal level of human professional support for diverse young people; issues covered further in sections below.

### 2.2 Realtime personalised interventions – ‘Mello’

A growing shift toward transdiagnostic models of mental illness has increased interest in shared mechanisms underlying multiple disorders (Dalgleish et al., 2020). One such mechanism is repetitive negative thinking (RNT)—a maladaptive cognitive process involving persistent, unhelpful thought patterns that sustain conditions like anxiety and depression (Moulds and McEvoy, 2025). Targeting RNT offers a scalable and personalised treatment strategy by addressing risk factors common across diagnostic categories (Dalgleish et al., 2020; Topper et al., 2017).

Smartphone-based interventions present a key opportunity for targeting mechanisms like RNT, enabling real-time, context-sensitive support when symptoms emerge (Luxton et al., 2011). Just-In-Time Adaptive Interventions (JITAIs) use real-time data to adapt intervention delivery based on an individual's state, environment, and behaviour, offering dynamic, personalised care (Nahum-Shani et al., 2018).

Applying the JITAI model, Mello was developed by Orygen Digital through a user-centred process involving young people and clinicians. In a pilot RCT with 55 participants experiencing clinical depression, anxiety, and RNT, *Mello* demonstrated moderate to large effect sizes for depression (d = 0.50), anxiety (d = 0.61), and RNT (d = 0.87), compared to a non-active control (Bell et al., 2023a). Mediation analysis indicated that reductions in RNT contributed to improvements in both depression and anxiety. The intervention was well-received, with 96 % of users recommending it, and 59 % still using the app at six weeks, exceeding typical engagement rates for self-guided interventions.

Qualitative findings highlighted Mello's value in prompting reflective responses during RNT episodes and supporting healthier cognitive habits (Valentine et al., 2024). Its flexible, self-guided format was also cited as enhancing accessibility. Some users provided feedback that the content could be better personalised to match their unique cultural, temporal and personal experiences. Importantly, these preliminary effects were achieved without clinical support, underscoring its potential for low-resource scalability. While further validation through larger trials is needed, Mello provides early evidence for the effectiveness of targeting transdiagnostic mechanisms in youth mental health. The app is currently available for free in Australia, with plans for international release.

### 2.3 Experiential digital mental health interventions

Extended Reality (XR) technologies, including Virtual Reality (VR), are increasingly being utilised as experiential DMHIs to support therapeutic outcomes across a range of mental health conditions (Bell et al., 2024). XR enables the creation of controlled, interactive environments where young people can safely engage in challenging therapeutic tasks via a headset. These interventions are typically grounded in established psychological frameworks such as cognitive behavioural therapy (CBT), mindfulness, and acceptance and commitment therapy.

A growing body of research demonstrates the effectiveness of VR-assisted therapy, particularly in treating anxiety disorders and psychosis (Wiebe et al., 2022). VR-based exposure therapy has shown significant reductions in anxiety symptoms and is considered non-inferior to in vivo exposure in adult populations (Morina et al., 2015; Schröder et al., 2023). In psychosis, VR interventions show promise in improving cognitive functioning, enhancing social skills, and reducing agoraphobic avoidance, hallucinations, and delusional thinking (Cella et al., 2022; Chan et al., 2023; Rus-Calafell et al., 2018). Importantly, individuals with psychosis report positive experiences and minimal adverse effects from VR therapy (Freeman et al., 2023). However, while generally safe, VR-based therapies can pose subtler risks that traditional safety protocols may overlook. Some users experience cybersickness, (nausea, dizziness, and headaches from sensory mismatch), despite hardware and software advances. Human factors like age and gender may still influence susceptibility. Others report dissociative experiences, such as feeling detached from thoughts or identity, especially in response to trauma or stress, with unclear long-term effects (Peckmann et al., 2022; Ramaseri Chandra et al., 2022; van Heugten-van der Kloet et al., 2018). Additionally, many interventions were developed without input from people with lived experience and often lack adaptation for the specific developmental needs of young people.

The launch of the Orygen Digital XR Innovation Lab (XR Lab) in 2021 marked a shift toward youth-focused VR therapies developed through co-design with young people. One key intervention, *REVIVE*, a VR-CBT package targeting social anxiety, was developed with young people with lived experience. While REVIVE VR-CBT therapy was found to be both feasible and acceptable for young people identified as being at Clinical High Risk (CHR) for psychosis, several significant barriers highlighted the need for a personalised and supportive implementation approach. For instance, some participants missed sessions due to difficulties in asserting themselves in everyday contexts, such as being unable to decline work shifts, request transportation, or communicate their needs within school or employment settings. Additionally, comorbid drug and substance use, busy schedules, and unforeseen life events further impacted engagement with the intervention (paper forthcoming). This was followed by *VISOR*, a semi-automated VR-CBT program focused on social cognition, incorporating theory of mind, emotion recognition, and bias modification. A large-scale RCT funded by the Wellcome Trust is underway to evaluate VISOR's effectiveness compared to traditional VR-CBT.

The XR Lab has also focused on developing VR-based third-wave interventions with a decentering focus, co-designed with young people and currently undergoing clinical evaluation. Additionally, a new VR-CBT adaptation is in development to target auditory and visual hallucinations, following qualitative interviews that highlighted their role in impairing recovery. A feasibility trial is planned for 2025. Another key development is *VR4VR*, a vocational recovery training program co-designed with young people to support employment readiness through integration with Individual Placement and Support services.

The integration of XR into mental health interventions is expected to expand significantly in coming decades (Fortune Business Insights, 2025). Ensuring that young people with lived experience are involved throughout development is critical to making XR therapies accessible, developmentally appropriate, and clinically meaningful. As the field advances, further research is needed to refine these technologies and ensure their ethical and equitable deployment in youth mental health systems (Bell et al., 2024).

## 3 Ten issues, challenges and opportunities in youth DMHIs

In over 15 years of co-developing DMHIs in partnership with young people and youth mental health systems, like others in the field, we have encountered both significant progress and persistent challenges that at times have either impeded advancement or resulted in unanticipated outcomes. Despite significant development, many of these challenges remain unsolved, reflecting the complex, interacting and multi-faceted systemic factors at play. Even so, the field has made considerable progress over the last 15 years, with many examples of successful DMHIs for youth being developed, trialled and less frequently, implemented, over this time (Balcombe and De Leo, 2023; Garrido et al., 2019). Below we describe the key challenges in the development, evaluation and implementation of DMHIs for young people we have directly encountered and noted in the literature, along with and our response to these challenges and opportunities for future improvement. While presented separately, we acknowledge that several themes recur across multiple challenges and are closely interrelated.

### 3.1 Meaningful co-design of digital products driven by lived experience that meet the diverse needs of both young people and multiple stakeholders

Developing effective and engaging DMHIs requires a deep understanding of young people's lived experience, the challenges they face, and the diverse and often competing priorities of other key stakeholders (de Beurs et al., 2017). Equally important is understanding the resources required to meet these needs, as well as the broader contextual and structural factors that contribute to shaping DMHI outcomes (Porche et al., 2022).

To ensure contextual relevance and alignment with real-world constraints, Orygen Digital has adopted co-design with lived experience and service stakeholders as a foundational development strategy (Malloy et al., 2023). This is supported by a multidisciplinary team, including clinicians, peer and vocational workers, researchers, designers, engineers, content creators, and business staff, who facilitate engagement and continuous feedback loops with young people, MOST and external service staff, partners, and funders. Operational investment is necessary to sustain this collaborative structure and to avoid disciplinary and project silos that may undermine long-term viability.

Central to this approach is the Lived Experience Participation Program (‘the Program’), which keeps young people at the heart of intervention design, development, and evaluation. The Program includes: (1) a youth advisory group (10–12 members, two-year tenure); (2) project-based co-design groups for specific initiatives; and (3) a flexible network for short-term engagements. These components offer varying levels of involvement, enabling a broad range of young people to engage in reciprocal and meaningful impact to DMHI development. The Program complements ongoing user research with those accessing, or choosing not to access, live DMHIs.

Lived experience involvement spans a spectrum from consultation to consumer-led projects (Colder Carras et al., 2023). Orygen Digital draws on best practice principles such as early engagement, power-sharing, lived experience leadership, capacity-building, and participatory methods, guided by published frameworks (Health Consumers Queensland, 2017; Kelly et al., 2017; National Institute for Health Research, 2019; Roper et al., 2018; Yang and Dibb, 2020). These have been adapted and integrated with human-centred design and agile methodologies.

Human-centred design fosters empathy and novel solutions through iterative prototyping and creative collaboration (Bazzano et al., 2017), while agile development supports rapid delivery of lean solutions and continuous refinement through real-world feedback (Beck et al., 2001). Though both methods are user-focused, they typically focus on problem-solving rather than power-sharing. Co-design and co-production principles address this by ensuring users (both young people and clinicians) influence decisions directly. Integrating co-design into agile workflows can be challenging due to differences in timelines and processes. Orygen Digital has developed an adaptive model that merges agile and human-centred design with co-production principles, enabling the creation of digital solutions that are responsive, collaborative, and relevant to young people and their clinicians.

Effective engagement of young people in agile projects requires the early establishment of a participation strategy, ideally led by designated lived experience staff. Models may include youth advisors embedded in teams, lived experience leadership in decision-making, or bespoke advisory and co-design groups. The Program takes an intersectional approach to engaging with lived experience perspectives, aiming for inclusion across mental health experiences, views, cultural identity, age, gender and sexuality, disability and digital access is essential to advocate for the needs of all intended users. Accessibility and inclusion are foundational. For example, MOST's national implementation engages high numbers of Aboriginal and/or Torres Strait Islander young people, LGBTIQA+ youth, and those from culturally and linguistically diverse backgrounds (Alvarez-Jimenez et al., 2024). Co-design with these populations strengthens cultural safety and relevance, enhancing engagement and effectiveness.

Participatory design has evolved from grassroots initiatives to a core tenet of DMHI development, reflecting growing awareness that meaningful collaboration with lived experience is essential to ensure DMHIs are relevant, inclusive, and effective (Jones et al., 2020). Yet this work must continue to deepen. Lived experience involvement should extend beyond individual intervention design into organisational strategy and governance to ensure alignment with real-world needs (Orygen, 2019), while keeping pace with rapid shifts in youth culture that shape expectations around digital experiences. This includes engaging underrepresented cohorts, families, carers, and all service personnel (The Wellcome Trust, 2024). Broadening co-design across systems will enhance support structures and better integrate digital mental health care into the environments in which young people live and seek help.

### 3.2 The challenge of sustained engagement in DMHIs

Engagement, defined as uptake, sustained use, and adherence (Lipschitz et al., 2023), remains one of the most persistent and consequential challenges in DMHIs (Fleming et al., 2018; Mohr et al., 2018; Valentine et al., 2025). This poses a significant theoretical challenge, as engagement is assumed to mediate and moderate clinical outcomes (Baumel, 2022). Yet, despite increasing sophistication in design and delivery, low uptake and poor retention continue to be an issue of concern in DMHIs (Torous et al., 2018; Valentine et al., 2025). Approximately 38.7 % of users discontinue interventions prematurely (Etzelmueller et al., 2020), and only 3.3 % of users of popular commercial mental health app (with a median of 100,000 downloads) remain active after 30 days (Baumel et al., 2019). Young people typically exhibit lower engagement than adults (Cross et al., 2022; Etzelmueller et al., 2020), even though they are digital natives (Ball et al., 2019) and endorse digital treatment options (Bell et al., 2022).

Multiple models have been proposed to capture its complexity. (Yardley et al., 2016) highlight the behavioural, cognitive and emotional dimensions of engagement, while (Perski et al., 2017) distinguish between the extent and the quality of engagement. Yet even as theoretical models strengthen design, the field still lacks standardised definitions and metrics for engagement. A recent review showed that 24 % of studies did not report engagement statistics, and those that did reported 25 distinct measures (Valentine et al., 2025). Without clarity on what engagement entails, or how it links to outcomes, it is difficult to scale effective design features or replicate success (Baumel, 2022). Furthermore, some evidence suggests that single session digital interventions can yield clinical improvements (Schleider and Weisz, 2017).

MOST integrates four complimentary frameworks to optimise engagement: Self-Determination Theory (SDT) (Ryan and Deci, 2017; Teixeira et al., 2020); Persuasive System Design (PSD) (Oinas-Kukkonen and Harjumaa, 2018); Supportive Accountability (SA) (Mohr et al., 2011) and the Digital Cumulative Complexity Model (DiCuCoM) (Cross and Alvarez-Jimenez, 2024). SDT offers a strong psychological foundation, proposing that engagement arises when autonomy, competence, and relatedness are supported, and (Teixeira et al., 2020) outlined 21 behaviour change techniques to operationalise SDT. SA predicts that engagement improves when users feel accountable to a supportive, trusted person (Mohr et al., 2011). PSD posits that digital systems can shape behaviour through design features such as reminders, tailoring, and social influence (Oinas-Kukkonen and Harjumaa, 2018), though evidence from a recent meta-analysis showed that its effectiveness in DMHIs is mixed (Valentine et al., 2025). DiCuCoM, drawing on ideas from Minimally Disruptive Medicine (May et al., 2009), conceptualises engagement as the dynamic interaction between the cognitive, emotional, or behavioural workload imposed by the DMHI and the user's capacity to manage it. It bridges behavioural science and motivational theory to explain why even well-designed interventions may fail to engage certain users. Empirical testing of this model is currently underway.

In practice, MOST uses specific engagement strategies inspired by these frameworks. Autonomy, competence, relatedness and accountability supported via self-paced content, scaffolded guidance, and peer connection, while personalised support fosters accountability (Mangelsdorf et al., 2024). Tiered content (Section 3.3) and varied levels of human support (Section 3.4) addresses treatment burden by matching intensity to user capacity. Digital storytelling contextualises strategies (De Vecchi et al., 2016), while multi-modal delivery enhances accessibility and reduces cognitive load (Liverpool et al., 2020).

We continuously refine our engagement strategies using data-driven analyses (Section 3.9). In the Horyzons trial, we went beyond simple login metrics to examine engagement patterns over time and across intervention components. Sustained engagement with both therapeutic and social networking elements was associated with improved outcomes in young people with early psychosis, while low use or reliance on the social network alone was less effective (O'Sullivan et al., 2022). In a subsequent study, the social network emerged as the most engaging component, often serving as a gateway to later therapeutic engagement (O'Sullivan et al., 2023). More recently, in our live transdiagnostic service, outcomes were predicted by different component usage patterns depending on symptom severity (Cross et al., 2025) (see Section 3.4).

Over time, we hope that through better integrating theoretical frameworks with real-time, data-informed usage patterns we can develop more engaging and therefore more effective interventions.

### 3.3 Toward more personalised and transdiagnostic digital therapeutic content

Personalisation is increasingly recognised as essential for sustaining engagement and improving outcomes in youth DMHIs (Borghouts et al., 2021; Garrido et al., 2019; Li et al., 2024; Liverpool et al., 2020). In face-to-face care, personalisation is standard, achieved through case formulation that integrates symptoms, goals, and psychosocial context (Langer and Jensen-Doss, 2018). In contrast, many DMHIs are designed for scale using static, population-level templates that can lack individual relevance and familiarity (Cross and Alvarez-Jimenez, 2024). Single-disorder models further limit relevance, failing to reflect the high comorbidity common in youth populations (Australian Bureau of Statistics, 2020-21; Lamers et al., 2011). This lack of tailoring reduces both engagement and therapeutic effectiveness (Baumel et al., 2019; Torous et al., 2018).

All Orygen Digital interventions are grounded in shared design principles: personalization, transdiagnostic psychological mechanism targeting, and alignment with clinical staging models (Cross et al., 2014; McGorry et al., 2006; Shah et al., 2020). This unified framework conceptualises mental ill health along a continuum and supports tailoring interventions to a young person's stage of need, symptom profile, and functional context.

MOST exemplifies this approach by delivering self- and clinician-guided transdiagnostic “therapy journeys” composed of 5–6 modules targeting shared mechanisms such as cognitive biases, avoidance, and emotion dysregulation (Clark, 2013; Dimidjian et al., 2011; Hayes et al., 2006). These journeys draw on techniques from second- and third-wave CBT traditions including CBT (Beck, 2011), ACT (Hayes et al., 1999), DBT (Linehan, 1993), MBCT (Segal et al., 2018), and the Unified Protocol (Barlow et al., 2017; Ehrenreich et al., 2009). Delivery formats range from fully self-guided to clinician-supported, including blended care, enhancing flexibility and scalability. Clinicians can personalise therapy journeys by reordering or selecting modules based on case formulation. Additional contextual content (e.g., on body image, loneliness, or system navigation) is also available further supporting user autonomy and relevance. A newly funded clinical trial will extend this model by testing how psychological mechanisms interact with user characteristics to guide more precise, individual-level care (Alvarez-Jimenez et al., 2025).

Other Orygen Digital interventions build on this shared foundation with distinct focal mechanisms. The Mello app targets repetitive negative thinking (RNT), a transdiagnostic process central to anxiety and depression (Moulds and McEvoy, 2025). Ongoing VR-based programs are designed to target specific mechanisms including social cognition (VISOR), social functioning (Revive), and decentering (Bell et al., in press). These reflect a process-based therapy (PBT) paradigm, which tailors treatment based on individual psychological processes rather than diagnostic categories (Hayes and Hofmann, 2021; Sanford et al., 2022).

Evidence for both transdiagnostic and process-based models is growing in youth (Bell et al., 2023b; Li et al., 2024). Meta-analyses indicate that transdiagnostic protocols are as effective as, or superior to, disorder-specific treatments (Li et al., 2024), particularly for managing comorbidities, and more feasible to implement at scale (Barlow et al., 2017; Newby et al., 2015, Newby et al., 2016; Păsărelu et al., 2017). Other large-scale DMHI trials have similarly demonstrated the promise of these approaches in real-world settings (Black et al., 2018; Dalgleish et al., 2020).

In sum, personalisation that is anchored in transdiagnostic, process-based, and stage-informed frameworks may further enhance the relevance, accessibility, and effectiveness of DMHIs for young people. Future work is underway to test and refine these models.

### 3.4 Optimizing the intensity of human support in DMHIs

Determining the appropriate type and intensity of human support in DMHIs remains a critical but unresolved issue. What intensity of human professional support, delivered by what modality, supports which sub-types of young people? Meta-analyses focusing on youth populations (Venturo-Conerly et al., 2022) indicate that DMHIs incorporating human support (whether synchronous or asynchronous) are associated with improved outcomes in anxiety, depression, and behavioural disorders compared to unsupported or self-guided approaches. Furthermore, studies in adults that also include older adolescents and young adults (16+) by (Linardon et al., 2019) and (Werntz et al., 2022) suggest that guided interventions generally yield better engagement and clinical outcomes than self-guided tools. However, human support isn't necessary for all users to achieve good outcomes (Linardon et al., 2019), and there is no clear dose-response relationship between support intensity and outcome (Shim et al., 2017).

The answer might lie in better treatment matching. Evidence suggests guided interventions may be most beneficial for individuals with moderate to severe symptoms, while self-guided tools may suffice for those with mild presentations (Karyotaki et al., 2021). Our own youth service-level data supports this: for those with mild-moderate mixed depression and anxiety symptomatology, improvements were primarily driven by the combination of increased human support and higher rates of content use. For those with severe symptoms, significant improvements were observed but were not solely attributed to the amount of DMHI professional support provided or content or consumed, likely because these users were also receiving more intensive in-person treatment (Cross et al., 2025).

Theoretical frameworks reinforce the value of even low-intensity human support. As discussed in Section 3.2, SA (Mohr et al., 2011), SDT (Ryan and Deci, 2017) and DiCuCoM (Cross and Alvarez-Jimenez, 2024) all propose that even brief, personalised human interactions can promote accountability, autonomy, competence, and reduce treatment burden. These mechanisms may be particularly important for young people, who are often new to mental health care and may require added scaffolding.

Professional human support, however, is resource-intensive and poses scalability challenges, particularly given ongoing workforce shortages in youth mental health services (McGorry et al., 2024). As a result, there is a need to balance human resource constraints with engagement, cost, and clinical outcomes (Lattie et al., 2022; Werntz et al., 2023).

Adaptive, data-informed approaches offer a potential solution. In face-to-face care, stratified stepped care models match support intensity to clinical need, escalating or de-escalating based on response (Bickman, 2020; Cross et al., 2014; Cross and Hickie, 2017; Mohr et al., 2019). These models have shown promise in reducing symptoms and improving cost-effectiveness in traditional settings (Fletcher et al., 2021; Jeitani et al., 2024; Stiles et al., 2019), but their application in DMHIs is so far limited.

To address this, we recently revised our DMHI youth service model to align with stratified stepped care (Cross and Hickie, 2017). A triage tool matches users to one of four support levels: semi-automated supportive messages from a clinician with on-demand single-session support, weekly brief coaching, or intensive and flexible teletherapy. A supported decision-making framework integrates these recommendations with user preferences to generate a personalised care plan (Andersson et al., 2023). These levels are later adjusted in response to changes in symptoms, functioning, and satisfaction. Evaluations of these changes are currently underway.

### 3.5 The potential added value of peer support and social networks in DMHIs

Social networking interventions hold considerable promise in youth mental health care, particularly as young people increasingly seek information and support online (Ridout and Campbell, 2018). Adolescents and young adults with mental health challenges are among the most frequent users of social media (Abdel-Baki et al., 2017; Birnbaum et al., 2017; Gayer-Anderson and Morgan, 2013), highlighting the relevance of mental health–specific platforms tailored to this demographic. A systematic review by (Ridout and Campbell, 2018) found that social networking interventions were perceived as engaging, supportive, and user-friendly by young people aged 15–25 years. However, conclusions were limited by the preliminary nature of the studies reviewed, which primarily comprised exploratory or pilot studies. Larger and more robust evaluations are needed to establish clinical effectiveness.

Digital peer-to-peer interactions have shown potential to promote help-seeking behaviours (Lawlor and Kirakowski, 2014) and are commonly used by young people for shared health concerns, often in anonymous formats (Barak et al., 2008; Heisler et al., 2010). Peer support, in both face-to-face and digital contexts, has also been associated with reduced relapse rates in adults with psychotic disorders (Davidson and Guy, 2012; Hyde, 2001).

Evidence from MOST highlights both the potential and complexity of integrating social networking into mental health care. Therapeutic social networks can promote empowerment, connection, belonging, and normalisation (O'Sullivan et al., 2024; Valentine et al., 2020), but barriers such as social anxiety, internalised stigma, paranoia, confusion about online social norms, limited interactions, low motivation, privacy concerns, and a perceived lack of autonomy may limit engagement. These findings underscore the need for flexible, personalised social features with clear guidelines and user-controlled interaction levels. Similarly, the self-directed option of MOST's therapy component supports autonomy for some, but others experienced low motivation and choice overload and subsequently disengage, highlighting the need to balance autonomy with structure (Valentine et al., 2021). Quantitative findings provide further evidence that the social network drives engagement with therapeutic content, but that sustained use of both therapy content, and the social network is necessary for improved outcomes (O'Sullivan et al., 2022, O'Sullivan et al., 2023).

While social networking features in DMHIs hold promise, they raise important safety and implementation considerations. Peer support alone often lacks the clinical expertise required for managing risk or making therapeutic decisions (Hartzler and Pratt, 2011), making professional moderation essential. Evidence shows that moderated forums, particularly those facilitated by clinicians or trained peer workers, are associated with better outcomes, whereas unmoderated platforms can lead to harm (Naslund et al., 2016, Naslund et al., 2019). This is especially relevant given the growing concern about commercial social media platforms, which have been linked to increased depression, anxiety, and social withdrawal in vulnerable young people (Baker and Algorta, 2016; Lin et al., 2016; Lup et al., 2015; Nesi and Prinstein, 2015; Vannucci et al., 2017). These findings underscore the need for DMHIs to distinguish themselves from commercial platforms by incorporating purpose-built, clinically moderated social networks as safer and more therapeutically aligned alternatives; particularly as governments move to restrict social media access for younger users.

Social networking features in DMHIs show promise for enhancing engagement and fostering peer connection, but the clinical value of these social features is contingent on careful integration with therapeutic content and clinical oversight. While early findings are encouraging, the optimal structure, moderation, and management of these communities remain unclear (Alvarez-Jimenez et al., 2016). Further research is needed to refine models that safely and effectively harness social support to improve clinical outcomes.

### 3.6 Implementation and integration of DMHIs into in real-world services

Given the relative nascency of the near 30-year history of the digital mental health field, implementation has only recently gained prominence as a key consideration for research and practice (McGinty et al., 2024). The prevailing narrative that few examples of successful implementation exist (Graham et al., 2020) is even more accurate applied to DMHIs for youth. To date, implementation research has largely focused on mental health service and primary care settings (Berardi et al., 2024). In youth populations, prevention and early intervention predominate, largely through randomised trials in school (Beames et al., 2021; Werner-Seidler et al., 2025) and university settings (Taylor et al., 2024) rather than within clinical environments (McGinty et al., 2024).

In contrast, Orygen Digital's implementation efforts have focused on embedding DMHIs within brick-and-mortar youth mental health service settings. Our experience aligns with broader implementation literature in identifying common barriers, such as high staff turnover and fluctuating motivation, both among clinicians and service users, impacting adoption and sustained engagement (Berardi et al., 2024). Similarly, established enablers such as access to training and knowledge, as well as the compatibility of interventions with existing service processes, are crucial to success.

Unique insights have also emerged from our large-scale rollouts, where implementation is monitored using the Consolidated Framework for Implementation Research (Damschroder et al., 2022). The barriers and facilitators identified in this implementation reflect patterns observed in implementation research guided by the CFIR framework. Key facilitators included access to knowledge and information, opportunities for reflection and evaluation in relation to the platform, the availability of promotional materials, and the ability to tailor strategies to the specific service context. Conversely, barriers such as workforce shortages and instability, low relative priority within services, and limited motivation among young people were noted. These findings align with prior CFIR-based research (Damschroder et al., 2022) and underscore the framework's utility in capturing critical implementation determinants across diverse health care settings, including digital mental health interventions. Periodic assessment of service context and the ability to adapt strategies in response have proven essential for supporting sustained implementation. Notably, despite the digital nature of our products, the most effective implementation strategies have been primarily analogue and relational in nature.

A challenge unique to digital implementation for youth is the structural discontinuity between youth and adult mental health services, a well-documented point of vulnerability in global mental health systems (McGorry et al., 2013). As young people age out of youth mental health services, typically at 18, they often encounter a fragmented and less developmentally attuned adult mental health system, contributing to service disengagement and unmet clinical needs (Singh et al., 2010). In contrast, the ability to enable care continuity is a key advantage of DMHIs (Hollis et al., 2017). However, embedding youth-targeted interventions within adult services presents its own challenges. These services often operate across broad age ranges and may struggle to integrate youth-specific tools into generalised workflows, limiting clinician awareness and adoption. This misalignment reduces the potential reach and effectiveness of digital tools designed specifically for young people and their ability to bridge fragmented systems.

Finally, young people differ in their expectations for digital experiences, shaped by their life-long engagement with commercially developed platforms that prioritise seamless design, personalisation, and user engagement (Andersson et al., 2019; Korte et al., 2014; Torous et al., 2018) young people may find DMHIs underwhelming by comparison. These expectations no doubt extend to DMHIs, which are constrained by funding and rightly focus on meaningful clinical outcomes, which may result in misaligned expectations, presenting a serious implementation challenge (Baumel and Muench, 2016). This disconnect impacts implementation in several ways; it may reduce uptake, limit engagement, and lead to negative perceptions of the intervention, feedback that can act as a barrier to implementation just as positive user feedback can serve as an enabler. Addressing this challenge requires early and ongoing collaboration between researchers, developers, service providers, and young people to ensure that interventions are both clinically sound and user experience focussed.

### 3.7 Incorporating DMHIs into routine clinical practice – ‘blended care’

While psychological therapy is predominantly delivered face-to-face, DMHIs are emerging as complementary tools to enhance accessibility and flexibility in care (Andersson et al., 2019). This integration, known as “blended care” (Wentzel et al., 2016) involves using digital components such as psychoeducation, mood tracking, or skills training within, between, or outside of face-to-face sessions. These components may be used prior to, during, or following therapy to extend therapeutic reach and support continuity of care (Erbe et al., 2017).

Blended care offers a cost-effective approach by allowing certain therapeutic functions to be delegated to evidence-based DMHIs, thereby increasing service capacity without compromising quality (Bell et al., 2022; Erbe et al., 2017). For clinicians, this enables the reallocation of limited therapeutic time toward more complex, in-session therapeutic tasks requiring nuanced interpersonal interaction, while more structured or repetitive components (e.g., psychoeducation or homework) can be managed digitally (Newby et al., 2021). For young people, blended care can offer a more contemporary and engaging model of care that aligns with their digital habits and preferences (Bell et al., 2022; Newby et al., 2021). Additionally, blended approaches may help reduce dropout rates due to treatment burden by decreasing the number of required in-person sessions. For example, (Rasing et al., 2021) recently found that adolescents receiving blended care for depression attended one-third of the number of sessions compared to those receiving face-to-face CBT alone or treatment as usual, with comparable clinical outcomes, providing early evidence for improved cost-effectiveness.

The COVID-19 pandemic showed that logistical barriers to digital delivery can be rapidly addressed when needed (Nicholas et al., 2021). However, clinician confidence, training, and digital infrastructure remain key barriers, often more so than user acceptance. Young people reported greater satisfaction with telehealth than clinicians, most of whom planned to continue digital approaches post-pandemic (Nicholas et al., 2021).

Delivering psychological therapy digitally requires a distinct set of clinical competencies (Lattie et al., 2019). These include familiarity with new modes of communication (e.g., video, chat, or app-based interactions), adaptations in clinical formulation and intervention planning, and the ability to manage the therapeutic relationship without a physical presence (Buckman et al., 2021; Scott et al., 2023). For blended care to be delivered effectively, clinicians must receive structured training, ongoing supervision, and support to maintain fidelity to the intervention model (Hadjistavropoulos et al., 2019, Hadjistavropoulos et al., 2018; Lattie et al., 2019).

The MOST platform is designed to augment, not replace, existing service-based care (Alvarez-Jimenez et al., 2024; Cross et al., 2023a; O'Sullivan et al., 2024). Embedded within youth services and co-designed with young people and clinicians, its delivery requires core competencies including: 1) understanding DMHI function, use, and evidence base; 2) assessing DMHI suitability for diverse users; and 3) delivering effective therapy via multiple digital modalities (e.g., phone, video, SMS, chat); and 4) navigate the ethical and clinical safety considerations of digital care, including privacy and digital literacy, monitoring for potential adverse events, deterioration, disengagement, misuse of peer support (Rozental et al., 2018). Ongoing work at Orygen Digital focuses on developing safe and effective training, supervision, and fidelity tools tailored to digital delivery. Co-design with clinicians can help ensure resources are feasible and aligned with real-world practice. As blended care models mature, youth-specific, evidence-based clinical guidelines will be essential to ensure effectiveness across diverse service settings.

### 3.8 Sustaining and scaling real-world DMHIs

Over the past decade, several DMHIs have moved from promising pilot studies to more mature, scaled solutions embedded in healthcare systems. Despite this success, the long-term sustainability and scalability of the majority of DMHIs remains a significant challenge. While the potential of DMHIs to increase access, reduce mental health service pressures, and improve mental health outcomes is widely recognised, translating this promise into real-world impact is frequently hindered by structural, financial, and systemic barriers.

Our own experience in sustaining and scaling DMHIs in real world contexts have been strongly aligned with the insights outlined in the Wellcome Trust's *Catalytic Environments* report (Batty et al., 2024), Rock Health's market analysis of innovation in youth mental health (Drasser et al., 2024), and strategic models for non-profit sustainability from the *Stanford Social Innovation Review* (Gugelev and Stern, 2015).

A persistent barrier to scale is the well-documented ‘research-to-implementation gap’. As noted by the Wellcome Trust, most academic and research settings are designed to optimise the production of novel knowledge, not its translation into practice (Batty et al., 2024). Incentive structures tend to prioritise novel innovation and publication over long-term implementation, resulting in a proliferation of well-studied but underutilised digital tools and interventions. Wellcome posits that addressing this challenge requires the cultivation of ‘catalytic environments’; organisations or ecosystems that actively bridge research, policy, and practice (Batty et al., 2024). Whether situated within universities, healthcare systems, or independent ventures, these entities facilitate sustained implementation by providing the cross-sector scaffolding necessary to develop, test, and adapt interventions within and for real-world contexts. At Orygen Digital, we deliberately integrate design, research, implementation, and policy translation functions to support the long-term sustainability of our digital offerings.

A second critical barrier relates to the lack of sustainable funding models. While billing mechanisms for digital care are advancing in some regions, reimbursement remains limited. In the U.S., for example, reimbursement is generally available for real-time telehealth but not for asynchronous digital services such as DMHIs (Graham et al., 2020). In contrast, countries like Australia and several parts of Europe have successfully integrated internet-based CBT into public mental health services through government and universal health insurance (Productivity Commission, 2020; Titov et al., 2018). However, even in these contexts, funding streams for more flexible or youth-specific digital models remain limited. Venture capital interest in youth mental health has grown substantially, with youth-oriented solutions representing 34 % of digital behavioural health investment in 2023, up from 15 % in 2018 (Drasser et al., 2024). Yet many start-ups struggle to secure follow-on funding or to integrate into existing service systems. This results in a “bridge to nowhere,” particularly for interventions developed within public or academic institutions that lack the infrastructure for independent commercial scale.

Traditional scale-up strategies, which focus on growing a central organisation or replicating a standardised model, may be ill-suited to the complexity of DMHIs. Some advocate for a shift from “scaling up” to defining an organisational “endgame”; the role an organisation aims to play within a broader solution (Gugelev and Stern, 2015). For many DMHIs, this may involve embedding within government-run services. For others, impact may be sustained through licensing, open-source dissemination, or partnerships with service providers. At Orygen Digital, we see the future of our DHMIs not necessarily as a product of organisational expansion, but as a sustainable and embedded component of the Australian youth mental health system, potentially supported through universal healthcare funding and public-private partnerships.

To achieve scale, DMHIs must be designed not only for efficacy, but also for integration. This involves early co-design with service users and clinicians (Section 3.1), alignment with care pathways (Section 3.6), and adaptation to policy, funding, and cultural contexts. Implementation strategies must account for variation across health systems and user populations, particularly in low- and middle-income countries where resources and infrastructure may be limited. Funders, too, have a critical role in this ecosystem. The Wellcome Trust and the Telstra Foundation offer promising examples of philanthropic organisations investing in both early-stage innovation and the more difficult, often under-resourced work of scaling, iterating, and embedding digital solutions.

Public-private partnerships may also offer new avenues for sustainable implementation. Collaborations between universities, governments, non-profits, and industry can help align financial incentives with clinical and social value, particularly when supported by long-term funding commitments and shared accountability frameworks (Kramer and Pfitzer, 2016). Success should be defined not only by the scientific evidence the DMHI generates, but by the systemic value delivered and long-term societal impact.

### 3.9 Gathering evidence, monitoring value and continuously improving DMHIs based on real-world data

For any healthcare system, data is foundational to continuous quality improvement (Enticott et al., 2021). However, mental health systems worldwide have historically lagged behind other areas of healthcare in the standardisation, collection, and utilisation of data, particularly in relation to measuring service quality and outcomes (Kilbourne et al., 2018; Lora et al., 2017). Although clinical data has significant potential to drive quality improvement across multiple domains of mental health care, including safety, timeliness, effectiveness, and equity (Bauer et al., 2014; Cross and Hickie, 2017; Kilbourne et al., 2018; Lora et al., 2017; Mohr et al., 2017), the data landscape in mental health remains highly fragmented and siloed (Smith et al., 2023). This fragmentation limits the ability to monitor care pathways, evaluate outcomes, and ultimately optimise service delivery.

Although digital by nature, DMHIs face similar data challenges. While they can generate rich, continuous data from multiple sources, in practice, information is collected across disconnected systems (such as platforms, health records, surveys, and administrative databases) at various points in care. Without structured data architecture and infrastructure, these data remain siloed, limiting their value for real-time monitoring, feedback, and quality improvement.

To address this, substantial investment in both specialist expertise and enabling technology is required. At Orygen Digital, we have built a multidisciplinary data infrastructure incorporating data engineers, software developers, clinical informaticians, data scientists, and behavioural machine learning specialists. These teams work collaboratively with clinicians and researchers to design scalable data pipelines that support both batch processing (scheduled data uploads for retrospective analysis) and near real-time data streaming (continuous data ingestion for dynamic decision support).

Data governance and integration are ongoing processes. Data ingested from varied systems must be standardised, structured, and mapped according to schemas that enable interoperability and consistency (Palojoki et al., 2024; Rabbi and Lamo, 2019). Once standardised, data can be connected to business intelligence tools to support real-time analytics or enable the application of machine learning algorithms for predictive modelling. This infrastructure enables systems to adapt dynamically based on new insights, supporting both clinical decision-making and system-level optimisation.

Technical sophistication alone, however, is insufficient. When DMHIs are embedded within traditional mental health services, technical integration often lags clinical integration (see Section 3.6). Many services still lack the interoperability required to link data across platforms, creating silos even within individual care pathways (Palojoki et al., 2024). Without person-level data linkage across systems, it becomes impossible to accurately evaluate DMHI outcomes or generate insights to inform policy and funding decisions (Baker et al., 2025; Cross et al., 2019; Rosenberg et al., 2015; Torous and Baker, 2016). This is a significant limiting factor in demonstrating to funding stakeholders the real value of DHMIs within broader health systems, reducing their potential for sustained impact.

A potential solution lies in the development of a ‘Learning Health System’ (LHS), frameworks that embed data-driven research and feedback loops into healthcare delivery to support ongoing improvement (Enticott et al., 2021; Tenenbaum and Ranallo, 2021). LHS's aim to “learn from every patient, every time” by leveraging routinely collected data to inform decisions at both clinical and system levels. This approach contrasts with the traditional reliance on RCTs as the primary mechanism for evidence generation. While RCTs remain the gold standard for evaluating clinical and cost-effectiveness (Hariton and Locascio, 2018), they are expensive, time-consuming, and often limited in generalisability due to strict inclusion criteria and controlled non-representative conditions.

Learning Health Systems (LHSs) enable the systematic use of real-world data and adaptive research designs for rapid, iterative knowledge generation, such as A/B testing, stepped-wedge, and micro-randomised trials (Klasnja et al., 2015; Mohr et al., 2017). These methods align well with digital contexts, where interventions evolve quickly, and user feedback is abundant. AI and machine learning further enhance this by detecting patterns, predicting outcomes, and tailoring interventions in real time (Bickman, 2020). However, leveraging these capabilities requires more than technical capacity, it demands investment in infrastructure, skilled workforce, and system-wide coordination.

Creating data-driven, learning-oriented environments is essential to scale DMHIs and integrate them meaningfully into complex mental health systems. By embracing LHS principles and moving beyond static evidence models, the field can advance toward more personalised, scalable, and responsive care.

### 3.10 Preparing for large language models in DMHIs

AI technologies, including large language models, are transforming digital mental health, offering potential to reduce clinician burden (Blease and Torous, 2023), improve personalisation (Dehbozorgi et al., 2025) and treatment precision (Bickman, 2020), support service navigation (Noble et al., 2022), guiding clinical decisions (Graham et al., 2019) and automating therapy (Durden et al., 2023; Hirschberg and Manning, 2015). However, their use in clinical contexts is in its early phases and there remains complex clinical governance and ethical issues to overcome (Cross et al., 2024; D'Alfonso, 2020; McCradden et al., 2023; Torous and Topol, 2025; Valentine et al., 2023).

Our initial work in this area is focussed on responsible early-stage exploration. In a survey we recently conducted (Cross et al., 2024) one-third of community members reported using AI for mental health purposes, with nearly half of those using AI as a personal therapist. Among clinicians, about half had used AI for research and documentation. While most found AI helpful, both groups expressed concerns around loss of human connection, ethical issues, data privacy, medical errors, potential misuse, and inadequate regulation (Cross et al., 2024).

Despite the immense promise, we and other experts remain cautious about the use of unsupervised, autonomous AI in clinical contexts due to serious risks. Recently, the eating disorder chatbot ‘Tessa’ had to be withdrawn after providing harmful advice (Chan et al., 2022). Crucially, LLMs do not reason, they predict the next word based on patterns, often giving the illusion of understanding, which can be misleading and dangerous if users overestimate the LMMs capabilities (Jiang et al., 2024; Mirzadeh et al., 2024).

Furthermore, the capacity for genuine empathy and compassion in LLMs is debatable. While they can mimic empathy (Ayers et al., 2023), their capacity for compassion (Gilbert, 2020), alliance repair, or context-sensitive intervention is limited (Morrow et al., 2023). For example, dialectical processes, like knowing when to challenge and when to validate, are fundamental to psychotherapy. While significant advances have recently been achieved through fine-tuning to align LLMs with human preferences, this approach can promote overly agreeable or sycophantic responses (Raedler et al., 2025) which can ultimately undermine therapy effectiveness.

Referring to the various use cases outlined in our survey (Cross et al., 2024), we have identified some promising areas of development and are actively co-developing these options with clinicians and lived experience representatives, mindful of these and other limitations. We see great promise in “collaborative AI” approaches where humans remain ‘in the loop’ to ensure safety and quality (Stade et al., 2024). For instance, a safer starting point for chatbot development could involve simulations: LLM “clients” interacting with AI “therapists” to model key indicators of when to shift strategies and how to repair ruptures. Promising early evidence supports the validity of synthetic therapy dialogues (Qiu and Lan, 2024). Alternatively, AI could generate decision support tools to guide human therapists—similar to how machine learning currently informs treatment planning based on client characteristics (Van Bronswijk et al., 2021).

As AI and LLMs are increasingly integrated into digital mental health interventions, robust quality assurance and ethical implementation frameworks will be essential. Emerging guidance, such as that proposed by (Löchner et al., 2025) provide important principles for building trustworthy, scalable, and ethically grounded digital interventions. These include transparency, accountability, human oversight, and risk management principles that align with our internal approach to early-stage AI development.

## 4 Conclusion

Over the past 15 years, Orygen Digital has accumulated a wealth of real-world experience in the development, testing, and implementation of youth-focused digital mental health interventions, albeit in high-resource settings. These experiences have highlighted both the immense potential and persistent complexities of delivering effective, scalable DMHIs. Rather than offering prescriptive global recommendations, this paper synthesizes key insights drawn from iterative practice, lived experience engagement, and emerging research. To strengthen the field, future work must move beyond traditional efficacy trials and focus on generating real-world evidence that captures complexity, context, and continuous improvement. By combining best practices in user-centred design, psychological science, context-adaptive implementation frameworks, business sustainability, and data-driven feedback loops, the field can accelerate the development–evaluation–implementation–scale cycle, ultimately increasing the value and impact of DMHIs in transforming youth mental health care.

The following is the supplementary data related to this article.

Supplementary Table 1 MOST evidence base (as of June 2025). Supplementary Table 1

## Declaration of competing interest

The authors declare the following financial interests/personal relationships which may be considered as potential competing interests: The authors are involved in the development, testing or implementation of Orygen Digital products, but beyond employment, derive no other personal or financial benefit from them.
