# Supplementary material for: Insights from fifteen years of real-world development, testing and implementation of youth digital mental health interventions
Source: Internet Interv. 2025 Jun 27;41:100849. doi: 10.1016/j.invent.2025.100849 (PMC12268945; doi:10.1016/j.invent.2025.100849)
Supplement: Supplementary Table 1 — MOST evidence base (as of June 2025). [file mmc1.docx]

# MOST Evidence Base (as of June 2025)

| **Paper and Reference Link** | **Summary of Findings** |
| --- | --- |
| Cross et al. (2025).  **Prediction of clinical improvement in a multicomponent digital mental health intervention for young people**.  *Beh Res and Therapy.* <https://doi.org/10.1016/j.brat.2025.104703> | - This study used machine learning to identify predictors of clinical improvement among 1,479 young people using the MOST platform across five Australian states. - Clinically significant reductions in depression and/or anxiety were observed in 43.8% of users over an average of 5.6 weeks of engagement. - Key predictors of improvement included human moderation and engagement with therapy and social networking features. - The predictive model achieved an area under the curve (AUC) of 0.72, indicating good discrimination between improvers and non-improvers. - Strongest effects of MOST for the 'Mild-Moderate' group. - Severe group still showed significant improvement, driven by a combination of MOST use *and* external service treatment. |
| Dwan-O’Reilly, M., Harrington, S., Gavin, C., Godfrey, E., Cowman, M., Gleeson, C., Sinnott, A. O. M., McCormack, J., Frawley, E., Burke, T., O’Connor, K., Birchwood, M., Heary, C., Alvarez-Jimenez, M., & Donohoe, G. (In Press). **Moderated Online Social Therapy (MOST) In Help-Seeking Young People: A Pilot Randomised Controlled Study.** *JMIR Preprints*. <https://doi.org/10.2196/preprints.73269> | - This pilot RCT assessed the feasibility and acceptability of MOST among help-seeking young people aged 16–25 in Irish youth mental health services. - Forty-four participants were randomised to either MOST + treatment as usual (TAU) (n = 24) or TAU alone (n = 20) for 8 weeks. - Engagement was high, with participants in the MOST group logging in a median of 7 times, and 83% accessing content or using interactive features. - Statistically significant improvements were found in social and occupational functioning (SOFAS: η² = 0.11, p = 0.03) and wellbeing (WEMWBS: η² = 0.09, p = 0.05) in the MOST group compared to TAU. - The intervention was rated positively: 87% found it helpful for understanding mental health, and 83% would recommend it to others. No adverse events were reported. |
| Alvarez‐Jimenez, et al. (2024).  **A national evaluation of a multi-modal, blended, digital intervention (MOST) integrated within Australian youth mental health services.**  *Acta Psych Scand****.*** <https://doi.org/10.1111/acps.13751> | - 6,589 young people from 262 youth mental health services. - Engaging, with 55.5% of users still using at 6 weeks and 40.1% at 12 weeks. - 94% of users would recommend MOST. - There was a statistically significant, moderate improvement in depression and anxiety at 12 weeks as measured by the PHQ4 (*d*=0.41). - Large treatment effects observed for young people on service wait lists (*d=*0.77). - 1,118 young people provided written feedback, of which 99% was positive and suggestions for new features. |
| O'Sullivan, et al. (2024).  **A Novel Blended Transdiagnostic Intervention (eOrygen) for Youth Psychosis and Borderline Personality Disorder: Uncontrolled Single-Group Pilot Study**. *JMIR Ment Health*, *11*, e49217. <https://doi.org/10.2196/49217> | - Evaluation of MOST as a blended tool used by face-to-face clinicians from youth mental health services. - 33 young people and 18 clinicians tested a blended model of MOST for psychosis and borderline personality disorder. - 93% of participants recommended MOST (eOrygen). - Significant moderate to large pre- to postintervention improvements for 9 (82%) of 11 outcome measures. - No adverse events or worsening of outcomes reported. - Young people reported peer support and personalised content help them engage. - Clinicians reported trustworthy content, strong evidence-base and positive platform reputation motivated use. |
| Engel L, et al. (2023).  **The cost-effectiveness of a novel online social therapy to maintain treatment effects from first-episode psychosis services: results from the Horyzons randomized controlled trial.**  *Schizophr Bull.* <https://doi.org/10.1093/schbul/sbad071> | - MOST- HORYZONS intervention cost $4789.59 less than TAU and led to better social functioning (20% cost reduction). - From a healthcare sector and societal perspective, costs were significantly lower in the MOST- HORYZONS group. - When young people had sustained engagement, costs decreased further, with a 46% cost reduction. |
| Cross, S., Nicholas, J., Mangelsdorf, S., Valentine, L., Baker, S., McGorry, P., Gleeson, J., & Alvarez-Jimenez, M. (2023). **Developing a Theory of Change for a Digital Youth Mental Health Service (Moderated Online Social Therapy): Mixed Methods Knowledge Synthesis Study**. *JMIR Form Res*, *7*, e49846. <https://doi.org/10.2196/49846> | - This study aimed to develop a comprehensive theory of change (ToC) for the MOST digital youth mental health service by synthesizing 15 years of clinical research, lived experience input, and implementation learnings. - The mixed-methods approach involved a narrative synthesis of clinical trial evidence, consultation with 22 stakeholders (including young people, clinicians, and researchers), and thematic analysis of platform usage and feedback. - The ToC identified six core components of MOST: therapy journeys, real-time clinician and peer support, social networking, curated content, safety protocols, and system integration. - Key change mechanisms were mapped to outcomes including improved engagement, wellbeing, help-seeking, connectedness, and clinical recovery. - The resulting model supports strategic planning, evaluation, and adaptation of MOST across diverse contexts and provides a foundation for future service co-design and scale-up efforts. |
| O'Sullivan, S., van Berkel, N., Kostakos, V., Schmaal, L., D'Alfonso, S., Valentine, L., Bendall, S., Nelson, B., Gleeson, J. F., & Alvarez-Jimenez, M. (2023). **Understanding What Drives Long-term Engagement in Digital Mental Health Interventions: Secondary Causal Analysis of the Relationship Between Social Networking and Therapy Engagement.** *JMIR Mental Health*, *10*(1), e44812. | - This study examined causal relationships between social networking use and engagement with therapeutic content within the Horyzons platform, using data from 170 young users. - Results showed that increased social networking activity significantly predicted subsequent engagement with therapy content (β = 0.11, p = .04), suggesting a reinforcing effect. - The relationship was bidirectional, but the predictive power of social networking on therapy engagement was stronger than the reverse. - These findings support the integration of social features in DMHIs to enhance engagement with therapeutic components over time. - The study highlights the importance of peer-to-peer interaction as a driver of sustained engagement in long-term digital interventions. |
| Pokowitz EL, et al. (2023).  **User experiences of an American-adapted moderated online social media platform for first-episode psychosis: Qualitative analysis**.  *Digit Health.* [https://doi.org/10.1177/20552076231176](https://doi.org/10.1177/20552076231176700) | - **USA**: Autonomous use of MOST was related to inter/intrapersonal factors as well as platform features in 20 users. - Perceived competence in social settings and managing MH was increased. - Experiences with peer workers and peers on platform promoted confidence in social settings. - Users provided feedback for improvement. |
| van Doorn M, et al (2023).  **The Effects of a Digital, Transdiagnostic, Clinically and Peer-Moderated Treatment Platform for Young People With Emerging Mental Health Complaints: Repeated Measures Within-Subjects Study.**  *JMIR Mhealth Uhealth;11:e50636* <https://mhealth.jmir.org/2023/1/e50636> | - **Netherlands**: 131 young people with MH symptoms. - Psychological distress decreased and psychosocial functioning improved over time with large effect sizes (P<.001 in both cases; ηp 2=0.239 and 0.318, respectively). |
| Lal, S., et al. (2023).  **Digital health innovation to prevent relapse and support recovery in young people with first-episode psychosis: A pilot study of Horyzons-Canada***.*  *Schizophr 9, 21* <https://doi.org/10.1038/s41537-023-00352-1> | - **Canada**: Majority of users provided positive feedback on their general experience (85%), found the platform easy to use (95%) and felt safe using it (90%). - 65% of users logged in at least 4 times over 8 weeks. - There was an increase in social functioning and no deterioration on the Clinical Global Impression Scale, indicating safety and acceptability. - No adverse events were reported. |
| Gleeson, J., Koval, P., Zyphur, M., Lederman, R., Herrman, H., Eleftheriadis, D., Bendall, S., Cotton, S. M., Gorelik, A., & Alvarez-Jimenez, M. (2023). **A randomized controlled trial of moderated online social therapy for family carers of first-episode psychosis patients in a specialist treatment setting**. *Schizophrenia research*, *255*, 203-212 | - This study evaluated “Altitudes,” a digital intervention based on the MOST model, designed to support family carers of young people receiving treatment for first-episode psychosis (FEP). - In a two-arm RCT (n = 94 carers), participants received either Altitudes plus treatment as usual (TAU) or TAU alone over a 3-month period. - Altitudes significantly reduced perceived stress among carers compared to TAU at post-intervention (Cohen’s d = 0.50, p = 0.03). - Improvements were also observed in carer self-efficacy and satisfaction with support, though effects on expressed emotion and burden were mixed. - The findings support the acceptability and efficacy of digital peer- and clinician-moderated interventions for improving outcomes in families of young people with psychosis. |
| Gleeson, J., Lin, A., Koval, P., Hopkins, L., Denborough, P., Lederman, R., Herrman, H., Bendall, S., Eleftheriadis, D., & Cotton, S. (2023). **Moderated online social therapy for carers of early psychosis clients in real-world settings: cluster randomized controlled trial.** *JMIR Mental Health*, *10*(1), e47722. | - This study tested “Altitudes,” a MOST-based intervention for carers of young people with early psychosis, delivered within real-world early intervention services across 14 sites. - The cluster-randomised trial included 148 carers and compared Altitudes + TAU versus TAU alone over a 6-month period. - Significant improvements were observed in carers’ perceived social support (p = 0.04) and confidence in supporting their young person (p = 0.03) in the Altitudes group. - No significant differences were found for perceived stress or burden, although trends favoured the intervention group. - The study demonstrates the feasibility and utility of implementing MOST-based carer interventions within public youth mental health systems. |
| Alvarez‐Jimenez, et al. (2021).  **The Horyzons project: a randomized controlled trial of a novel online social therapy to maintain treatment effects from specialist first‐episode psychosis services.**  *World Psychiatry, 20*(2), 233–243.  <https://doi.org/10.1002/wps.20858> | - 170 users randomised to Horyzons-MOST or TAU. - MOST group were 5.5 times more likely to find employment or enrol in education than those in the TAU group over 18-months. - 47% engaged for at least 9 months. - MOST group had half the rate of visits to emergency services (19% vs. 39%) and hospital admissions (13% vs. 27%) compared to the TAU group over 18-months. |
| O'Sullivan S, et al. (2022).  **Characterizing Use of a Multicomponent Digital Intervention to Predict Treatment Outcomes in First-Episode Psychosis: Cluster Analysis.**  *JMIR Ment* *Health;9(4):e29211* <https://mental.jmir.org/2022/4/e29211> | - Of 86 users, identified 3 use profiles. - Higher use of more components showed significant improvements in social functioning, negative symptoms, and overall psychiatric symptom severity compared to other profiles and TAU group. |
| Gleeson J,. et al. (2021).  **A pilot trial of Moderated Online Social Therapy for family and friends of young people with Borderline Personality Disorder features**.  *Early Interv Psychiatry.* [doi.org/10.1111/eip.13094](https://doi.org/10.1111/eip.13094) | - 20 adult carers of 10 young people with Borderline Personality Disorder (BPD). - Improvements were observed in carers in areas like burden, stress, expressed emotion, family communication, quality of life, functioning, coping, and knowledge of BPD. |
| Valentine, L., et al M. (2021).  **Young people’s experience of online therapy for first‐episode psychosis: A qualitative study.**  Psychology and Psychotherapy: Theory, Research and *Practice*, *95*(1). <https://doi.org/10.1111/papt.12356> | - Online therapy experiences for 12 first-episode psychosis varied among users. - Unique benefits included on-demand help-seeking and positive distraction, potentially contributing to fewer emergency department visits and a trend towards fewer hospitalisations in the intervention group. |
| Alvarez-Jimenez, et al (2020).  **A Novel Multimodal Digital Service (Moderated Online Social Therapy+) for Help-Seeking Young People Experiencing Mental Ill-Health: Pilot Evaluation Within a National Youth E-Mental Health Service.**  *Journal of Medical Internet Research, 22(8), e17155*. <https://doi.org/10.2196/17155> | - 157 users of MOST+. - High user ratings on ease of use, relevancy, helpfulness, and overall experience. - 98% of participants had a positive experience, 82% felt better, 86% felt more socially connected, 92% would recommend it. - Significant improvements in 10/11 outcomes assessed. - Significant correlations between system use, perceived helpfulness, and several secondary outcome variables. |
| Rice, S., et al. (2020).  **Leveraging the social network for treatment of social anxiety: Pilot study of a youth-specific digital intervention with a focus on engagement of young men*.***  *Internet Interv. 20: p. 100323*.  <https://doi.org/10.1016/j.invent.2020.100323> | - 89 male users of MOST-Entourage. - Significant improvements in social anxiety symptoms, depression, belongingness, social connectedness, and reduced loneliness among young males. - 98.6% of participants would recommend MOST-Entourage to others with social anxiety. |
| Bailey, E., et al. (2020).  **An Enhanced Social Networking Intervention for Young People with Active Suicidal Ideation: Safety, Feasibility and Acceptability Outcomes*.*** *Int J Environ Res Public Health. 17(7).* <https://doi.org/10.3390/ijerph17072435> | - 20 young people at risk of suicide. - The study found significant and reliable improvements in self-reported outcomes, including a reduction in suicidal ideation, among participants. - The enhanced online social networking intervention, MOST-Affinity, was found to be safe, feasible, and acceptable for young people experiencing active suicidal ideation. |
| Ludwig, K.Aet al. (2020).  **Horyzons USA: A moderated online social intervention for first episode psychosis*.***  *Early Interv Psychiatry.* <https://doi.org/10.1111/eip.12947> | - **USA**: 26 users of MOST-Horyzons with psychotic disorders. - Participants showed notable improvements in psychosis-related symptoms, negative emotions, depressive symptoms, and loneliness. - Was found to be feasible and well-tolerated, with a high retention rate (92.3%) and significant participant engagement (79.2%). - Feedback suggested improvements such as a smartphone app, larger community, and private messaging options. |
| Lal, S., Gleeson, J., Rivard, L., D'Alfonso, S., Joober, R., Malla, A., & Alvarez-Jimenez, M. (2020). **Adaptation of a Digital Health Innovation to Prevent Relapse and Support Recovery in Youth Receiving Services for First-Episode Psychosis: Results From the Horyzons-Canada Phase 1 Study.** *JMIR Form Res*, *4*(10), e19887. <https://doi.org/10.2196/19887> | - This Phase 1 study tested the feasibility, acceptability, and safety of the adapted Horyzons digital platform for youth with first-episode psychosis (FEP) in Canada. - A total of 20 participants aged 18–35 were recruited from two early intervention services and used the platform for 8 weeks. - High levels of satisfaction and usability were reported: 80% said they would recommend Horyzons-Canada to others. - No adverse events were reported, and participants particularly valued the peer-to-peer support and expert moderation features. - The study confirmed the feasibility of implementing the Horyzons model in a Canadian context and informed a subsequent RCT. |
| Valentine, L., McEnery, C., O’Sullivan, S., Gleeson, J., Bendall, S., & Alvarez-Jimenez, M. (2020). **Young people’s experience of a long-term social media–based intervention for first-episode psychosis: Qualitative analysis.** *Journal of medical Internet research*, *22*(6), e17570. | - This qualitative study explored the lived experiences of 17 young people with first-episode psychosis who participated in a long-term intervention using the Horyzons platform. - Participants described MOST as a unique and valuable space for connection, self-expression, and guided therapy, particularly during isolation or recovery setbacks. - Key themes included a sense of safety and normalisation, the value of peer support, flexibility in engagement, and the importance of clinical moderation. - Users emphasised the role of the platform in enhancing self-understanding, reducing stigma, and providing practical coping strategies. - Findings underscored the potential of moderated digital social media interventions to foster sustained recovery and social connectedness in early psychosis. |
| McEnery, C., et al (2019).  **Social anxiety in young people with first-episode psychosis: Pilot study of the EMBRACE moderated online social intervention*.***  *Early Interv Psychiatry.* <https://doi.org/10.1111/eip.12912> | - 10 users with first-episode psychosis and social anxiety. - All participants viewed the intervention (MOST-EMBRACE) as positive and safe and would recommend the intervention to others. - Significant improvement in social anxiety symptoms (measured by SIAS and Liebowitz Social Anxiety Scale) in young people with persistent and long-standing social anxiety |
| Alvarez-Jimenez, M., et al. (2018). **Enhancing social functioning in young people at Ultra High Risk (UHR) for psychosis: A pilot study of a novel strengths and mindfulness-based online social therapy.**  *Schizophrenia Research, 202, 369–377.* <https://doi.org/10.1016/j.schres.2018.07.022> | - This was the first study to develop and test an online intervention (MOST-MOMENTUM) designed for ultra-high risk for psychosis patients with 14 users at risk of psychosis. - Significant large improvements in social functioning (*d*= 1.83, *p*< 0.001) and subjective wellbeing (*d*= 0.75, *p*= 0.03). - All participants had a positive experience, considered MOST safe, and would recommend it. - 93% found MOST-MOMENTUM helpful. |
| Rice, S. et al. (2018).  **Moderated online social therapy for depression relapse prevention in young people: pilot study of a “next generation” online intervention.**  *Early Intervention in Psychiatry*, *12*(4), 613–625.  <https://doi.org/10.1111/eip.12354> | - MOST-REBOUND to prevent relapse in youth depression in 42 users. - Significant increase in full remission rates: from 5 participants at baseline to 19 at 12 weeks (*p* < 0.001). - Improvement in depression scores (MADRS; *p* = 0.014, *d* = 0.45). - High system usage: 3,034 logins (avg. 72.2/user), 2,146 posts (avg. 51.1/user). - 84% (32 participants) rated the intervention as helpful. |
| Alvarez-Jimenez, M., Bendall, S., Lederman, R., Wadley, G., Chinnery, G., Vargas, S., Larkin, M., Killackey, E., McGorry, P. D., & Gleeson, J. F. (2013). **On the HORYZON: moderated online social therapy for long-term recovery in first episode psychosis.** *Schizophr Res*, *143*(1), 143-149. <https://doi.org/10.1016/j.schres.2012.10.009> | - This was the first study to pilot the HORYZONS platform, a moderated online social therapy intervention designed to support long-term recovery in young people following a first episode of psychosis. - The pilot involved 20 participants and demonstrated high engagement: 70% used the system for more than three weeks, and 95% used the social networking component. - No adverse events were reported; users described the platform as safe and supportive. - Participants reported increased social connectedness and empowerment. - Reductions in depression symptoms were observed after one month, suggesting preliminary clinical benefit. |
